# The Cold War in pharmacology: a bibliometric analysis of Berlin’s contributions to Naunyn‑Schmiedeberg’s Archives of Pharmacology (1947–1974)

**DOI:** 10.1007/s00210-024-03115-6

**Published:** 2024-05-17

**Authors:** Mert Erkan Basol, Roland Seifert

**Affiliations:** Institute of Pharmacology, Hannover Medical School, D-30625 Hannover, Germany

**Keywords:** Post-war Berlin, Bibliometric analysis, East-West-Berlin comparison, Naunyn-Schmiedeberg’s Archives of Pharmacology, Cold War

## Abstract

After World War II, Berlin was divided into the West, controlled by The United States, the UK, and France, and the East, controlled by the Soviet Union, resulting in a Cold War for decades. This bibliometric study analyzes the influence of the Cold War on pharmacological research in Berlin by evaluating publication patterns in Naunyn-Schmiedeberg’s Archives of Pharmacology from 1947 to 1974 (*n* = 383). The publications highlight the political disparities in scientific output, exacerbated by the founding of the Free University of Berlin (FUB) as a countermeasure to Soviet repression, promoting academic freedom in West-Berlin. Researchers in West-Berlin published many more papers in Naunyn-Schmiedeberg’s Archives of Pharmacology than researchers in East-Berlin and received much more citations. West-Berlin adopted English as a scientific language much more rapidly than East-Berlin. West-Berlin and East-Berlin focused on totally different research topics. This paper demonstrates how political freedom, financial support, and internationalization boosted research productivity in West-Berlin. In contrast, political suppression, financial scarcity, and restricted international ties hindered scientific development in East-Berlin.

## Introduction

Following World War II, Berlin emerged as a city in political tensions, with Germany divided into four zones and Berlin itself divided among the Allied powers (Nowobilska and Zaman 2014). This division caused the rise of divergent political ideologies, casting Berlin as a crucial ideological battleground (Scribner 2009). The city’s critical role highlighted the escalating conflict between the Soviet Union and the Western Allies, deeply shaped by opposing forces of “Americanisation” and “Sovietisation” (Plischke 1965; Nikel 2021).

The Soviet Union’s initial appropriation of all academic bodies in Berlin, coupled with US authorities’ initial neglect in educational policies, underscores the influence of political agendas on Berlin’s academic institutions. A US strategic pivot to advocate for the revival of academic liberty in West-Berlin culminated in the establishment of the Free University of Berlin (FUB) in 1948 (Herken 1999; Philippu 2004; Groot 2014). The US initiative to strengthen science and education sought to integrate Germany globally and position it as a defense against communism. The establishment and funding of the FUB represented a clear stance against Soviet authoritarianism, epitomizing the West’s commitment to academic freedom (Woetzed 1965; Marshall 1997; Herken 1999; Wolter 2004; Krige 2006).

The Soviet occupying forces’ dominance in university policies post-war extended to academic institutions across both East- and West-Berlin, despite the Western Allies’ focus on ‘denazification’ which initially overlooked this overreach. The Soviets, treating universities as ideological tools, imposed communist doctrines on Humboldt University of Berlin (HUB), stifling academic freedom and hindering progress (Woetzed 1965; Marshall 1997; Herken 1999; Krige 2006; Eickelpasch 2015; Sotiriadis 2015; Ahlers et al, 2023).

In a recent study, we analyzed the publications in Naunyn-Schmiedeberg’s Archives of Pharmacology from German pharmacological institutes in the period from 1947 to 1974 (Basol and Seifert 2024). Substantial disparities between West- and East-Germany emerged. This paper investigates Berlin’s pharmacological community within the same timeframe, extending the scope of our first study. This report presents the first systematic examination of the scholarly and research environment for pharmacology in Berlin against the backdrop of Cold War politics, offering a nuanced perspective on the city’s academic landscape.

## Materials and methods

### Extraction process and identification of publication data

Data for this study were extracted from the Naunyn-Schmiedeberg’s Archives of Pharmacology official website using Python and Beautiful Soup (Python Software Foundation 2021; Richardson, L. 2021; Springer 2024). The focus was on bibliometric analysis of publications from the post-war era, specifically from 1947 (volume 204) to 1974 (volume 286), resulting in 4839 publications. The analysis covered bibliometric details including publication type, titles, authors, affiliations, DOIs, issue dates, volumes, and citations. Emphasis was placed on original papers. Data organization was performed using the Pandas library in Python, and findings were cataloged in Excel (.xlsx) for visualization through graphs and tables.

### Data structuring and accuracy assurance

A Python unit test was carried out to check the accuracy of the data collected. The data in the Excel spreadsheet were then compared with the original information on the SpringerLink website (official site) to ensure the correctness of the data collection. 

### Publication activity

**Fig. 1:**
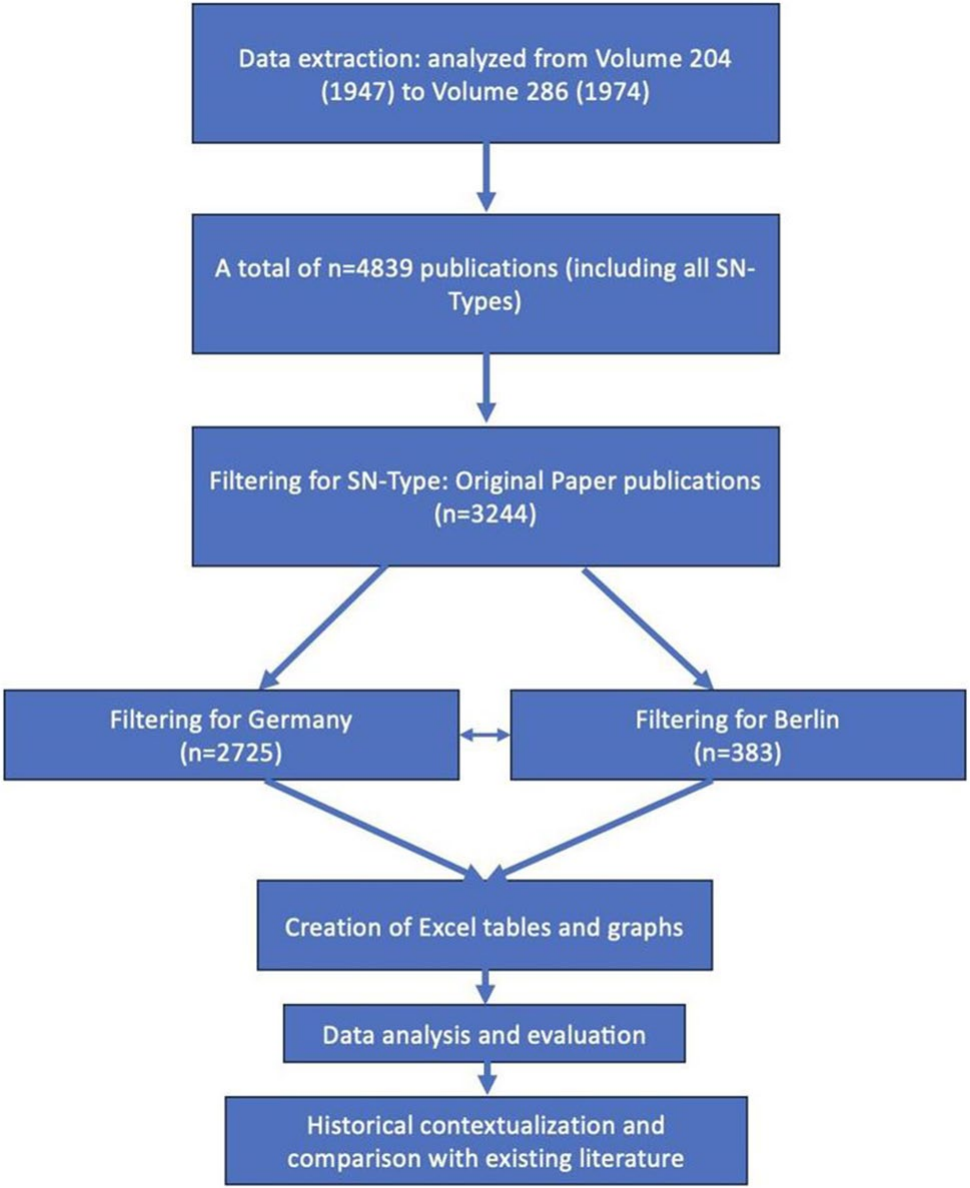
Flowchart representation of the analysis procedure

After filtering the data set to original papers (*n* = 3244), a further subdivision was made into publications from Germany (*n* = 2725) and Berlin (*n* = 383). The differentiation within Germany allowed a dichotomous representation of Western- and Eastern-Germany after the cities were categorized into “Western” and “Eastern” cities using Python matplotlib (Fig. 1). The same method was then used for the city of Berlin in the period from 1947 to 1974, after manually determining where the West-East border ran. The border was drawn based on information from the Berlin State Center for Political Education (BLpB 2014). With the help of this data, the publication activity of West- and East-Germany as well as West- and East-Berlin during the period from 1947 to 1974 could be depicted. Subsequently, using the population number (1974) and the publication activity between 1947 and 1974, the publication rate per million inhabitants was presented to make a statement about the “productivity” of certain geographical locations (Fig. 1).

In West-Berlin, scientific publications were concentrated in two institutions: The Institutes of Pharmacology at the FUB and Schering AG. In addition to this, there were other institutes such as the Biological Department of the Institute for Tumor Diseases at the Rudolf Virchow Hospital (RVH) and the Institute for Internal Medicine at the Hubertus Hospital (HH), whose contributions to the journal remained marginal. Over the investigated period, FUB had (for different time periods) up to four pharmacological institutes: (1) Institute of Pharmacology, (2) Institute of Clinical Pharmacology and Toxicology, (3) Institute of Toxicology and Embryopharmacology, and (4) Institute of Pharmacology and Toxicology of Veterinary Medicine. In this analysis, they are summarized under the term “Pharmacological Institute of the FUB”. In East-Berlin, the Pharmacological and Toxicological Institute of the Humboldt University of Berlin (HUB) was the focus of scientific publication activity. Other institutions in East-Berlin were not mentioned due to their low presence in the journal (Philippu 2004; Archiv der Freien Universität Berlin 2024).

### Language trends in the publications

To display the publication languages of the original papers, the Excel data were subjected to Python’s Langdetect module, which recognizes the language of the titles. The data were then compared concerning West-Berlin and East-Berlin and transferred to the period from 1947 to 1974.

### Citation analysis

The citations of the filtered publications were extracted individually for each publication using Python and Beautiful Soup from CrossRef, which is directly linked to the official journal website. CrossRef is updated daily. The citation data were last accessed on March 10, 2024.

### Subject areas

For the filtered publications (original papers), thematic priorities were manually assigned based on the title names. The main categories of pharmacology were taken from the textbook “Basiswissen Pharmakologie” and served as grouping criteria for the thematic classification of the publications (Seifert 2018). Although the main topics of the textbook covered most publications, some could not be categorized. Therefore, additional main topics such as “Purinergic system,” “Substance P,” and “Toxicology” were created manually. This thematic classification made it possible to identify trends in the period from 1947 to 1974 as well as geographical differences between West- and East-Berlin. The thematic analysis was then visualized using Excel in the form of pie charts.

### Authors

The author analysis included the examination of the first, second, and last authors of the filtered publications (original papers) from Berlin. This analysis revealed specific publication frequency trends of the authors and led to the identification of the 15 most influential authors of the journal in this period. Furthermore, additional bibliometric data were collected, which provided information on the citations of the works of the authors in question. We also investigated which institutes the authors belonged to and how research work was organized at these institutions. Thematic focuses were also examined.

## Results and discussion

### Publications

Berlin delivered 383 original papers. Of these, 328, or 85%, were published in West-Berlin and only 55, or 15%, in East-Berlin (Basol and Seifert 2024). This distribution underlines the dominant role of West-Germany and especially West-Berlin in the academic discourse. The prevalence of Western scientific publications within the journal implies that trends observed in Berlin serves as a microcosm for Germany as a whole (Basol and Seifert 2024).

The population of West-Berlin is about twice as high as that of East-Berlin, yet the publication rate of 155 original papers per million inhabitants in West-Berlin is far higher than in East-Berlin, where the rate is just 51 original papers per million inhabitants. These figures show that West-Berlin had a much higher publication productivity in relation to its population than East-Berlin (Basol and Seifert 2024).

**Fig. 2:**
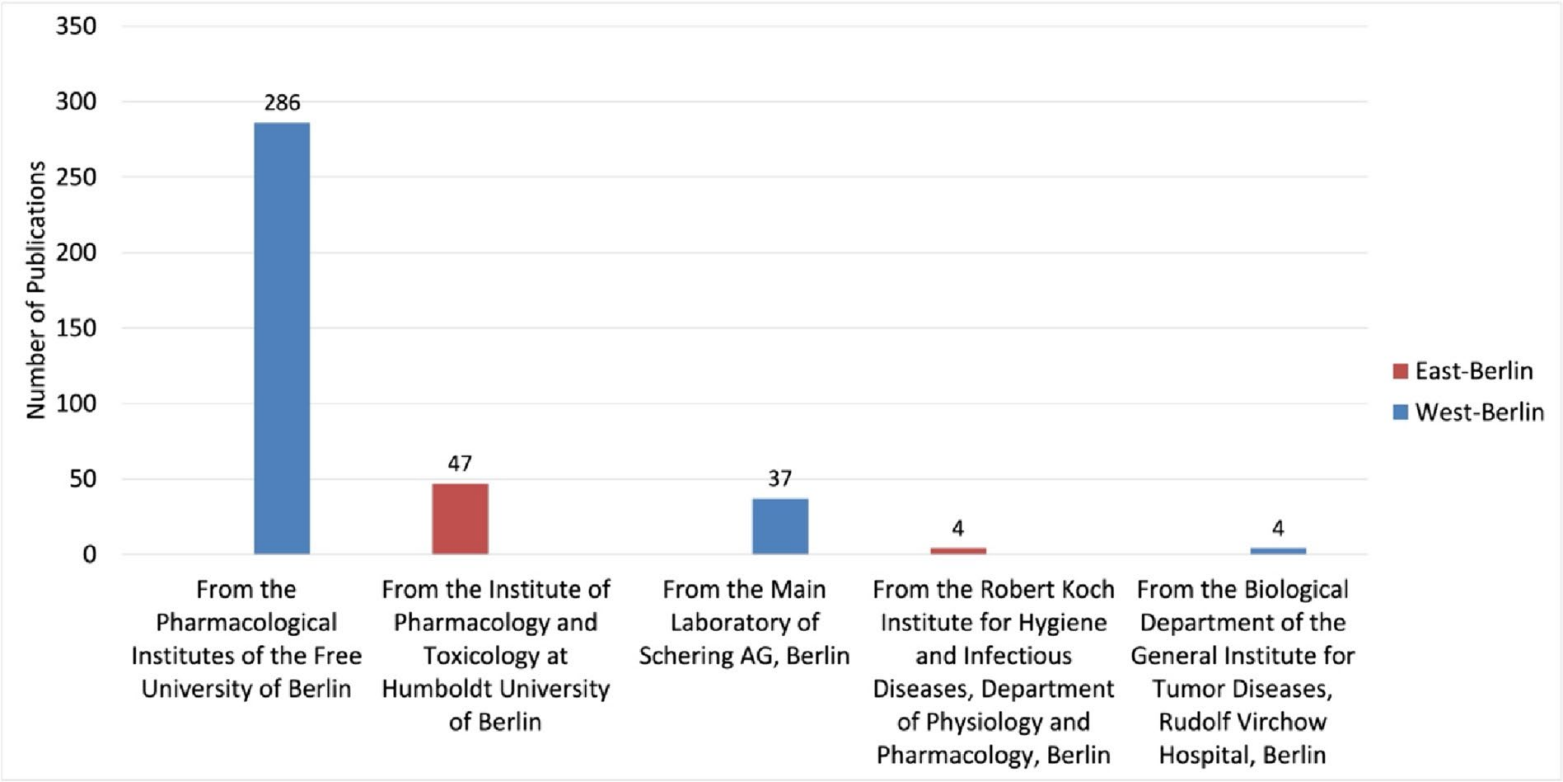
Pharmacological publication output (original papers) from West- and East-Berlin Institutes from 1947 to 1974—West-Berlin (blue) and East-Berlin (red)

The Institutes of Pharmacology at the FUB stand out with 286 publications, making up 87% of all West-Berlin-originated original papers (Fig. 2). In contrast, East-Berlin exhibits modest research activity, with the pharmacological institute at the HUB contributing 47 publications (original papers). Additionally, Schering AG in West-Berlin, with its 37 publications (original papers), underscores the vibrant research ecosystem in West-Berlin (Fig. 2).

**Fig. 3:**
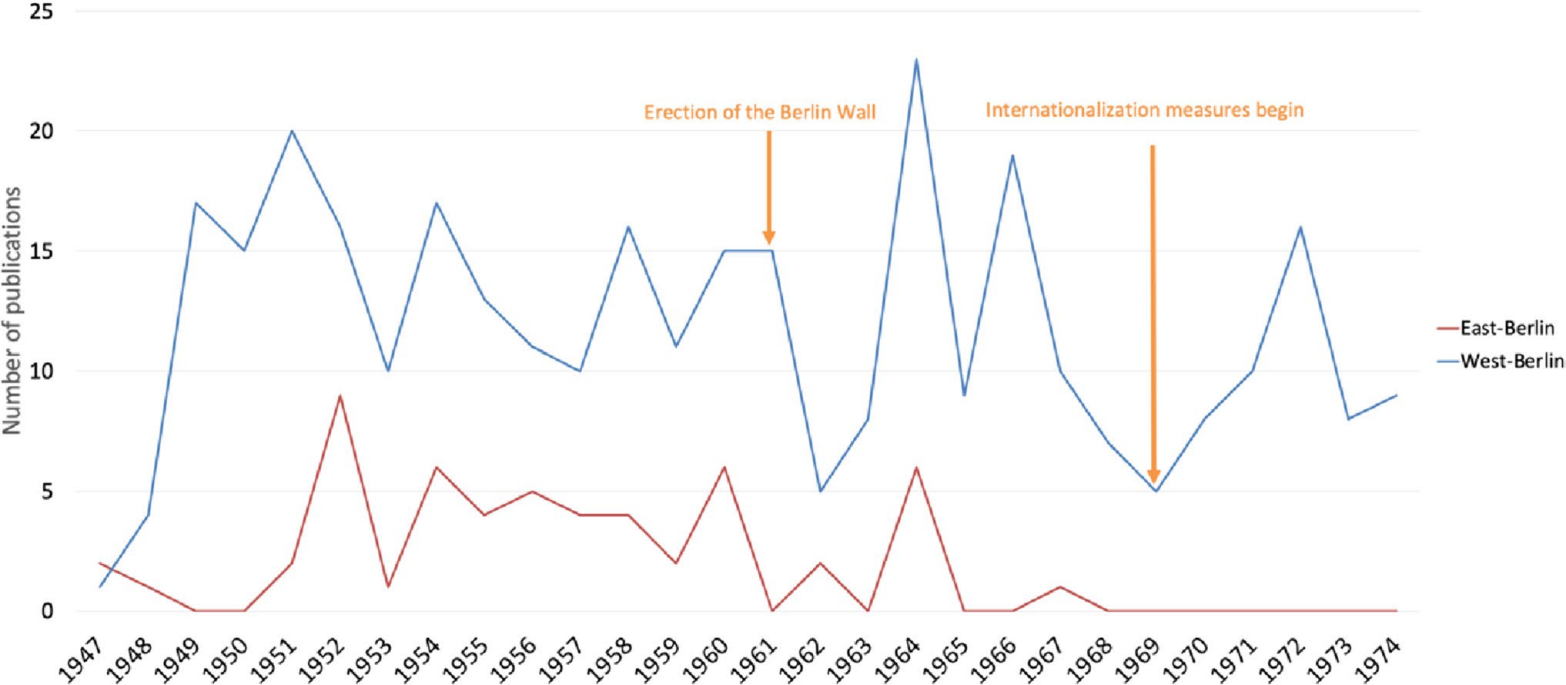
Publication trends of West- and East-Berlin Institutes from 1947 to 1974—West-Berlin (blue) and East-Berlin (red)

After World War II, Naunyn-Schmiedeberg’s Archives of Pharmacology experienced its first publication hiatus since its establishment in 1873 (Starke 1998; Fig. 3). The pharmacological institute at the University of Berlin (later HUB), extensively damaged during the war, serious immediate scientific challenges after World War II, further compounded by the Soviet occupation controls after its relocation to Dahlem (West-Berlin). This is evidenced by the mere four publications from the HUB’s pharmacological institute in 1947, and only five from Schering AG in West-Berlin by 1948. (Napoli 1949; Herken 1999; Philippu 2004; Krige 2006; Dats et al. 2023; Fig. 3).

**Fig. 4:**
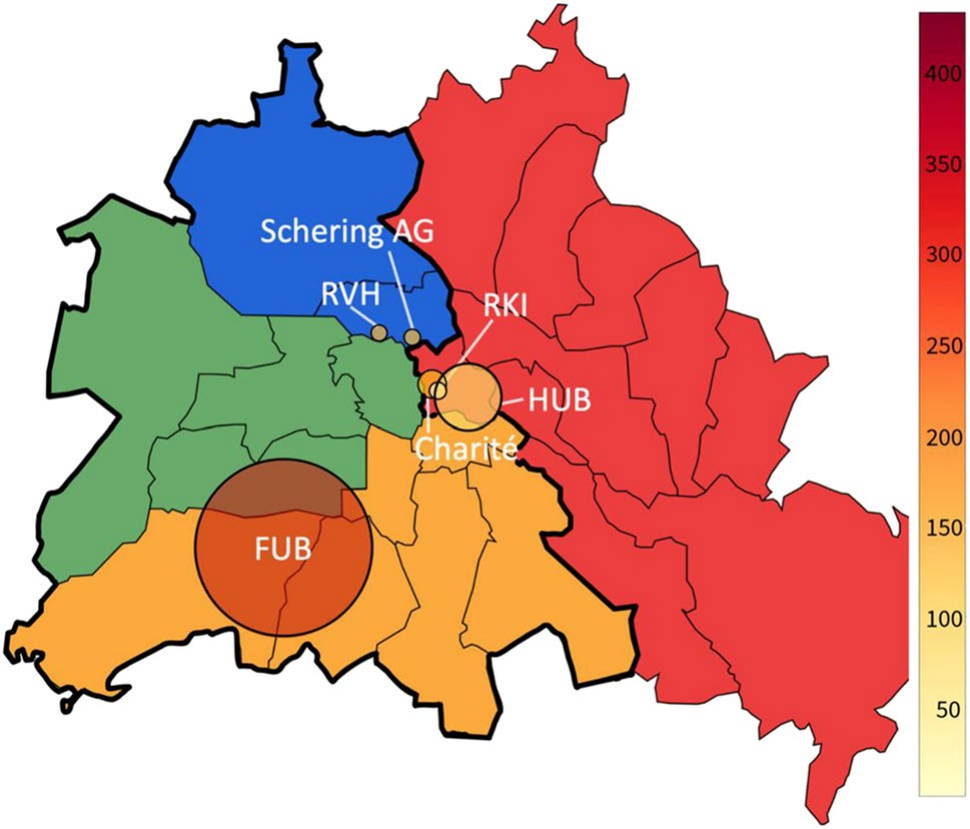
Pharmacological research in Berlin institutes: Zonal Allocation and scholarly output between 1947 and 1974. Charité: From the Main Medical University Clinic and Polyclinic of the Charité, Berlin (East-Berlin). RKI: From the Robert Koch Institute for Hygiene and Infectious Diseases, Department of Physiology and Pharmacology, Berlin (East-Berlin). HUB: From the Institute of Pharmacology and Toxicology at HUB (East-Berlin). FUB: From the Pharmacological Institutes of the FUB (West-Berlin). RVH: From the Biological Department of the General Institute for Tumor Diseases, Rudolf Virchow Hospital, Berlin (West-Berlin). The thick black line (green) encircles West-Berlin with the US sector (orange), the British (UK), and the French sector (blue). The soviet sector (East-Berlin) is labeled in red color. The number of papers from the various institutions is dually labeled with color intensity (right-hand column) and circle size. The approximate locations of the institutions are indicated by the circles in the Berlin map. Note that all institutions (except for the newly founded FUB) are very close to the border between West-Berlin and East-Berlin

After FUB’s inception in 1948, HUB experienced a publication hiatus, a result of pharmacologists’ migration to FUB. By 1950, FUB had produced 32 original papers, a stark contrast to HUB’s just three papers (Herken 1999; Philippu 2004; Fig. 3). The limited number of HUB papers shows that Soviet politicization of academic institutions in Berlin led to a decline in research activity (Nachmansohn 1979; Herken 1999; Krige 2006).

On September 22, 1948, in response, the Berlin Magistrate and the US sector moved to establish the FUB, officially founded in December, 1948, free from Soviet political oppression (Herken 1999; Philippu 2004; Groot 2014). In 1948, FUB contributed one original paper to the journal (Fig. 3).

The USA viewed scientific work and institutions as crucial for international cooperation and unity (Krige 2006). They aimed to reintegrate Germany into the international community, driven less by altruism than concerns over communism and the desire to instill Western values in the German population (Krige 2006; Sotiriadis 2015; Ahlers et al. 2023).

Establishing the FUB offered the USA an effective means to implement this strategy (Mason 1959; Krige 2006). The FUB received substantial support through funding programs like the Ford Foundation, providing 10 million Deutsche Marks (DM, German Mark), and 20 million DM from the Administration of the US Occupation Zone (Krige 2006; Archiv der Freien Universität Berlin 2024).

During this period, Berlin’s pharmacological research primarily tackled post-war health crises such as hunger edema and drug shortages. Schering AG was instrumental for the production of pharmaceuticals (Herken 1999; Kobrak 2002; Coppack 2022). From 1950 to 1957, the journal recorded 27 original papers from Schering AG (Fig. 2).

Despite Fritz Jung’s (1903–1981) appointment as HUB’s director in 1950 and the subsequent slow restoration of research facilities, political tensions between West- and East-Berlin impeded cooperation, reflected in the low publication output from HUB until the late 1960s (Catudal and Rush 1978; Herken 1999; Philippu 2004; Wunderlich 2010; Fig. 2).

The June 17, 1953, uprising in East-Germany, most notably East-Berlin, was triggered by the Stalinist economic program’s failure, primarily due to increased work norms (Ingimundarson 1994). Protesters not only demanded the overthrow of the SED (Socialist Unity Party of Germany) regime but also sought improved living and working conditions (Millington 2021). Ongoing reparation payments from East-Germany to the Soviet Union further fueled unrest, exacerbating resistance (Geerling et al. 2021). The SED regime tried to discredit the uprising by labeling it a “failed fascist coup”. (Millington 2021). This trend is evident in the reduced number of original papers published by the HUB from 1952 to 1963 (Mason 1959; Fig. 3).

The construction of the Berlin Wall in 1961 profoundly affected Berlin’s capacity to conduct research. In West-Berlin, which was geographically located in the Soviet occupation zone (East-Germany) but controlled by the West Allies, the limitations imposed by the Berlin Wall led to an initial decline in research and hindered international collaboration (Fig. 3). In East-Berlin, the erection of the wall signaled a shift in state-society relations, leading to increased political (pseudo) stability (Ross 2004). However, the closure of the border and subsequent division of the city had detrimental effects on the scientific community, resulting in the closure of research institutes and the loss of thousands of jobs for researchers (Koenig 1999). Additionally, the wall symbolized the Cold War divide and ideological differences between communism and democracy impacted the city on a broader scale (Harrison 2011).

A noteworthy occurrence took place in 1964 when six original papers by Kurt Repke (1919–2001) on cardiac glycoside pharmacodynamics and kinetics were published, marking a rare instance of work from East-Berlin institutes being featured in Naunyn-Schmiedeberg’s Archives of Pharmacology (Fig. 3). Despite the FUB’s initial aspirations to act as a collaborative bridge to East-Berlin’s academic community, the restrictions imposed by the Soviet occupation intensified East-Berlin’s scientific isolation, hindering potential cooperation (Catudal and Rush 1978; Nachmansohn 1979; Herken 1999; Krige 2006).

Since 1941, Springer has served as the publisher of Naunyn-Schmiedeberg’s Archives of Pharmacology (Starke 1998). Following World War II, the company established its first corporate headquarters in West-Berlin. This strategic placement facilitated post-war relations with the USA and contributed to Springer’s evolution as a Western-oriented publishing house (Sarkowski and Götze 1992; Starke 1998; Fig. 3). The process of internationalization was reinforced by the establishment of a subsidiary of Springer in New York in 1964 (Götze 1994). Such Western alignment likely contributed to the disproportionately low volume of publications originating from East-Berlin’s institutes during this period (Fig. 4).

Publication trends in West-Berlin until 1967 revealed around 12 original papers annually, indicating a period of relative stability (Fig. 3). Despite the FUB engagement in international collaborations, including fellowships and guest lectures, Naunyn-Schmiedeberg’s Archives of Pharmacology primarily featured articles in German, maintaining its identity as a predominantly German journal (Heubner 1953; Herken 1999; Heinsohn and Nicolaysen 2021).

However, a notable decline in publication activity began in 1967, due to the broader internationalization of the scientific community and a growing preference for publishing in English-language journals, associated with higher academic prestige (Francisco 2015; Di Bitetti and Ferreras 2017; Zehetbauer et al. 2022; Dats et al. 2023; Gzoyan et al. 2023). Following the publisher’s internationalization efforts in the late 1960s, there was a resurgence in the volume of publications from West-Berlin (Fig. 3) (Starke 1998). The annual output, which had dipped to about six original papers, rebounded to an average of 14 publications per year from 1970 to 1974 (Fig. 3). 

### Language

**Fig. 5:**
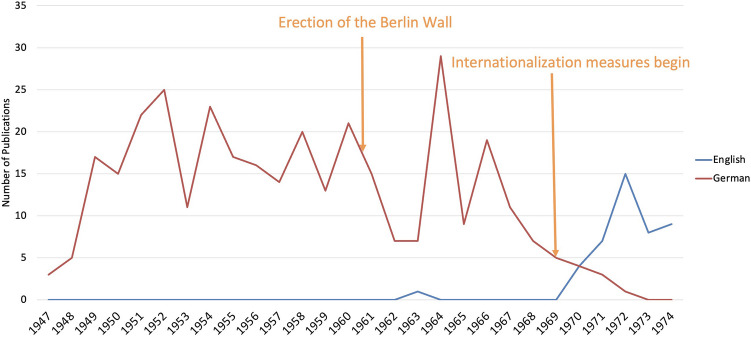
Publication language trends in pharmacological papers in Berlin from 1947 to 1974

Until the late 1960s, Naunyn-Schmiedeberg’s Archives of Pharmacology published in German, with a single English original paper appearing in 1963, reflecting efforts to re-establish the international reputation of German scientists after the war (Starke 1998; Krige 2006; Gzoyan et al. 2023; Ahlers et al. 2023; Fig. 5). From 1965 onwards, German publications (original papers) decreased, an indication of the influence of the international scientific community and the need to establish English as the lingua franca of the journal (Starke 1998; Hamel 2007; Phillipson 2009; Bajerski 2011; Billings 2015; Fig. 5).

In the early 1960s, German researchers increasingly targeted international journals, prioritizing global over national recognition (Gibbs 1995; Davydova 2020; Zehetbauer et al. 2022; Dats et al. 2023). Responding to these shifts, the journal endorsed English in 1969 onwords, mandating its use by 1973 and anglicizing its name in 1971. This strategic move enhanced the journal’s position in the pharmacological sciences (Starke 1998; Dats et al. 2023; Hattori and Seifert 2023). The rise in English publications from 1969, eventually surpassing German articles, underscores the importance of this strategic pivot. By 1972, English-language articles from Berlin reached 15 and just a single German contribution (Fig. 5).

**Fig. 6:**
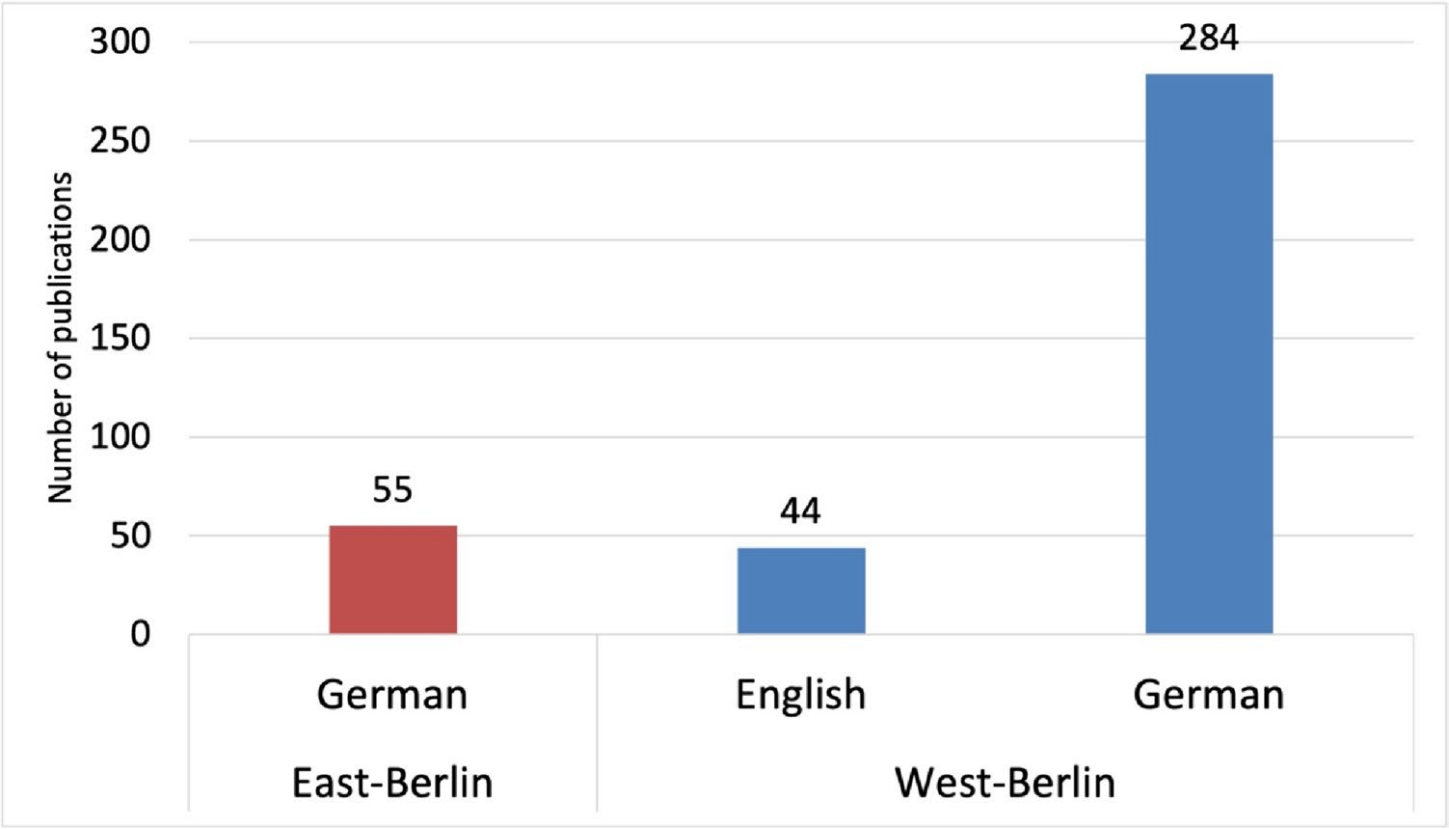
Comparative analysis of publication (original papers) languages between West- and East-Berlin Institutes. Total number of papers is shown—West-Berlin (blue) and East-Berlin (red)

This linguistic transformation solely occurred within West-Berlin’s pharmacological institutes (Fig. 6). Remarkably, 43 out of 44 English-language articles were published after 1969, with all contributions originating from the FUB (Figs. 5 and 6). This pattern reflects West-Berlin institutes’ support for English in the journal, capitalizing on established ties with Anglophone countries like the US and the UK towards the late 1960s (Heubner 1953; Starke 1998; Krige 2006).

In contrast, institutes in East-Berlin experienced a stagnation in publication activities after 1965, barely touched by the journal’s push for internationalization. The escalating Cold War isolation hindered East-German scientists from integrating into the Western scientific community (Paasi 2005; Krige 2006; Ahlers A. L., Hennings, J., & Schmidt, F. 2023).

The exclusion of East-German pharmacologists from the 1963 German Society for Experimental and Clinical Pharmacology and Toxicology (DGPT) meeting, which led to the establishment of a separate GDR society in 1967, illustrates that the internationalization by Western standards remained inaccessible to the Eastern Bloc during the Cold War (Fritz Markwardt 1995; Starke 1998; Paasi 2005; Krige 2006; Tsvetkova 2008).

Springer’s Western orientation, coupled with political dynamics, contributed to the academic divide, in the journal highlighting the contrast between the “free” environment of West-Berlin (represented by the FUB) and the restricted atmosphere of East-Berlin (represented by the HUB) (Mason 1959; Mueller 2004; Paasi 2005; Krige 2006; Hecht 2011; Archambault et al. 2017; Choi 2022)

### Citations

Citation metrics serve as an accepted measure for international recognition (Narin et al. 1991; Francisco 2015). The impact factor of journals and the citation rate of authors can be influenced by the country of origin of the publications. Works originating from authors and journals situated in authoritarian regimes tend to receive lower citation rates and diminished international visibility than papers from democratic countries (Glanzel 2001; Callaham 2002; Tahamtan et al. 2016). After World War II, Germany found itself self-inflictedly marginalized from the global research community, resulting in a significant knowledge gap that international collaborations sought to address (Herken 1999; Krige 2006).

Otto Krayer (1899–1982) made efforts to integrate the pharmacological institute of the HUB into global scientific dialogues but faced resistance from the Soviet authorities (Herken 1999). In contrast, the HUB’s branch in Dahlem (West-Berlin) thrived with support from the Unitarian Service Committee (USC), particularly following the establishment of the FUB (Heubner 1953; Herken 1999; Krige 2006). This facilitated a gradual narrowing of the knowledge gap for pharmacologists in West-Berlin (Herken 1999).

**Fig. 7:**
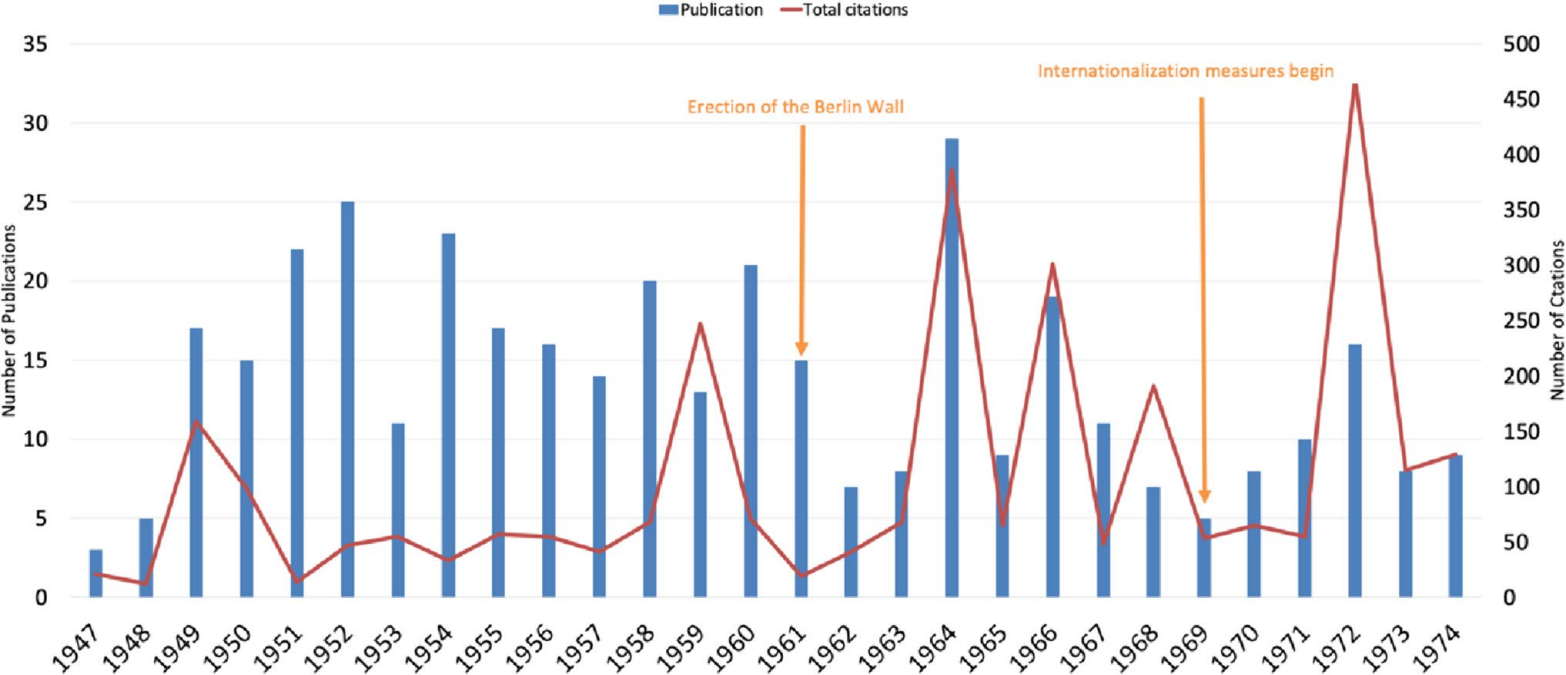
Trends in citations and publication numbers of original pharmacological papers (1947–1974) (last accessed 10th March 2024). Publication numbers are shown as blue bars; citation numbers are shown as red line

Citation numbers increased from 1951 to 1959, followed by a notable decline in citations thereafter. This decline correlates with increasing political tensions and the construction of the Berlin Wall in 1961 (Fig. 7). The period between 1970 and 1974 marks a notable increase in both publication and citation numbers, indicating the successful impact of the editors’ internationalization efforts. This uptrend not only signifies enhanced visibility but also suggests greater engagement with the international scholarly community, affirming the effectiveness of the strategies adopted (Starke 1998; Fig. 7).

**Fig. 8:**
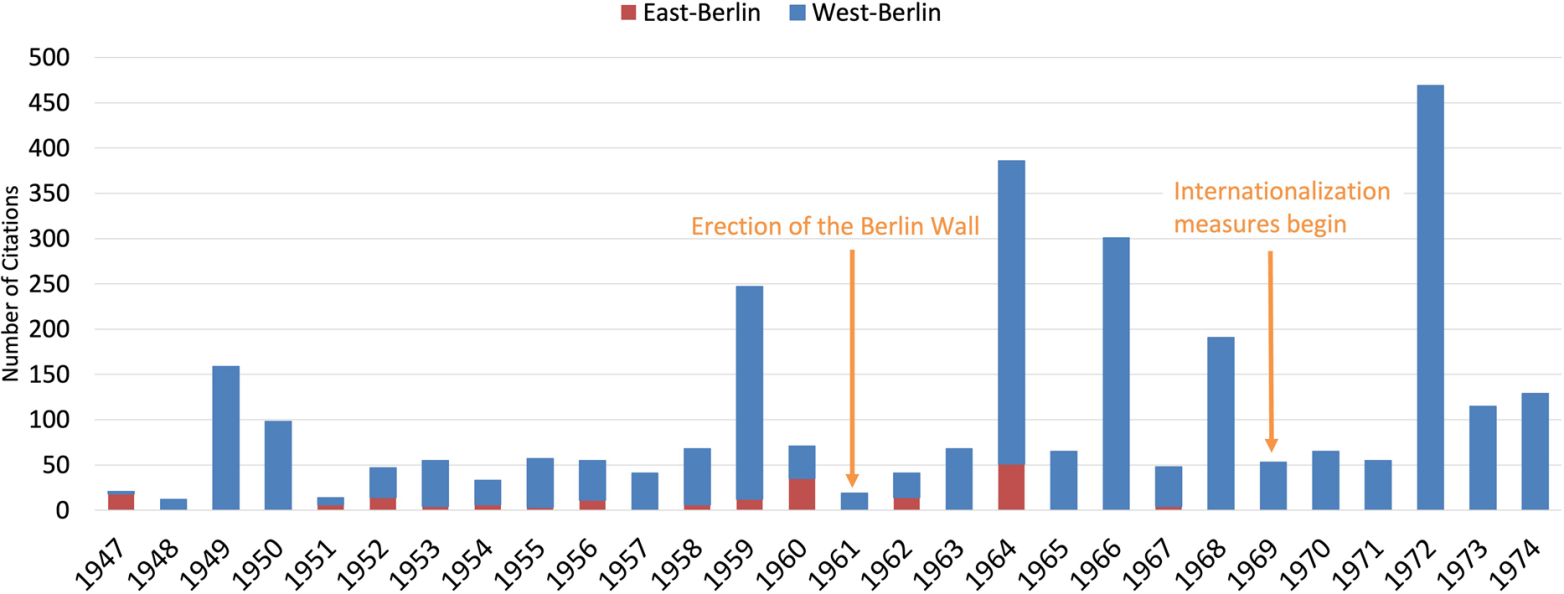
Citations of pharmacological papers from West- and East-Berlin: a comparative analysis (1947–1974) (last accessed 10th March 2024)—West-Berlin (blue) and East-Berlin (red)

**Fig. 9:**
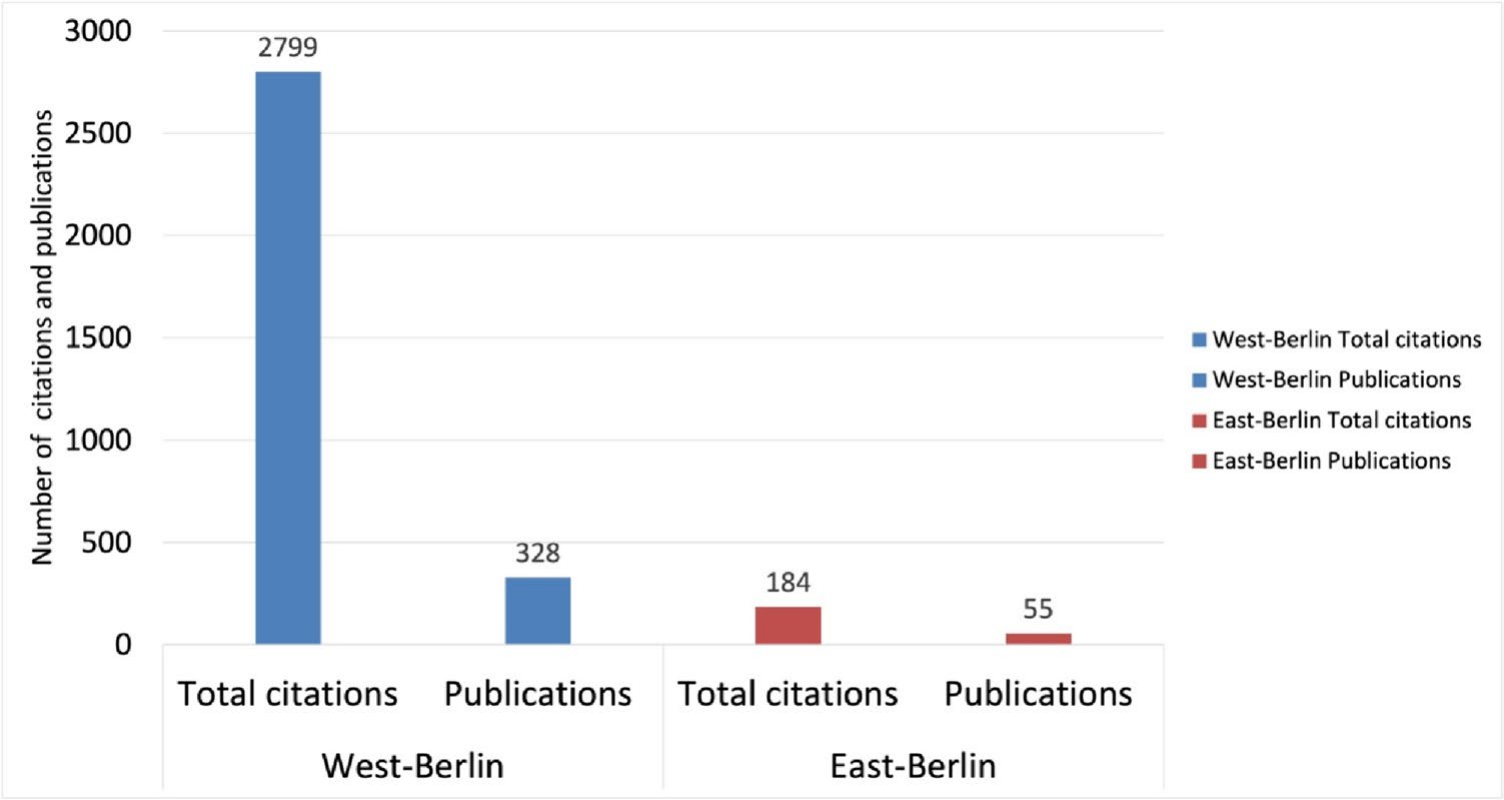
Comparative analysis of publication impact: Total Citations and publications (original papers) in West- vs. East-Berlin (last accessed 10th March 2024)—West-Berlin (blue) and East-Berlin (red)

Unlike FUB, the HUB continued to isolate itself, which led to it also isolating itself from many Western academic journals (Narin et al. 1983; Mason and Tent 1989; Herken 1999). This resulted in a stark contrast in publications and citations between East-Berlin and West-Berlin (Figs. 8 and 9). A notable rise in citations for West-Berlin followed the FUB’s founding, such as for contributions to hemoglobin and methemoglobin research by Manfred Kiese (1910–1983) (Fig. 7 and Table 1). These findings, pivotal in understanding hemoglobin physiology and addressing methemoglobinemia in war veterans, received many citations (Pechura and Rall 1993; Hagiwara and Inoue 2015).

**Table 1:**
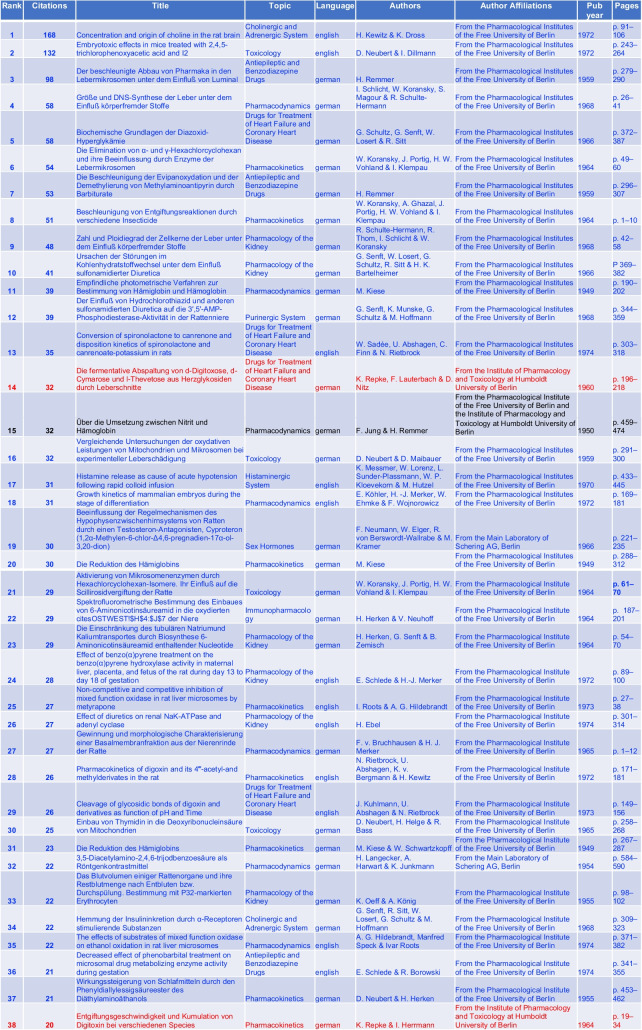
Top 38 most-cited pharmacological articles (original papers; last accessed 10th March 2024)—West-Berlin (blue) and East-Berlin (red). Note that one paper (15) was a joint publication from pharmacologists from East-Berlin and West-Berlin. This paper is marked in black

Herbert Remmer’s (Rank 3) research on Luminal’s pharmacokinetics and Diether Neubert’s (Rank 16) work on mitochondrial and microsomal oxidative performance in experimental liver damage garnered considerable scientific attention, accumulating 98 and 134 citations, respectively (Remmer 1959; Neubert and Dillmann 1972; Table 1). During this period, there was a focus on developing safer antiepileptic drugs and addressing clinical challenges such as alcohol-induced liver degeneration (Smith et al. 2007; Szabo and Mandrekar 2010; Brodie 2010).

Günter Schultz (Ranks 5, 10 and 12 in Table 1), working in the group of G. Senft, made highly cited contributions on the pharmacology of diuretics. Conceptually most important were his early studies on cyclic AMP (paper ranked on position 12 in Table 1). These studies laid the foundation for the field of signal transduction research in Germany (Philippu 2004). In 1983, Schultz followed Hans Herken as the Director of the Institute of Pharmacology of FUB.

In 1964, the FUB’s Institute of Pharmacology, led by H. Herken (1912-2003), achieved high citations (Fig. 8). Koransky’s research on γ-hexachlorocyclohexane (lindane) elimination (rank 6) and liver DNA synthesis under the influence of foreign substances (rank 4) obtained significant attention (Table 4). The research on lindane experienced popularity due to the desire to understand the carcinogenic effect of the compound (Philippu 2004; Vijgen et al. 2005; Fig. 8).

The publication and citation metrics from East-Berlin highlight a significant trend towards academic isolation, exacerbated by the construction of the Berlin Wall in 1961 (Baker 1993; Krige 2006; Gzoyan et al. 2023; Figs. 7 and 8). Despite relatively high citations for topics such as the detoxification rate of digoxin and the fermentative cleavage of d-digitoxose, d-cymarose, and l-thevetose from cardiac glycosides by Kurt Repke (Rank 14 and 38) in 1960 and 1964, the overall citation frequency for East-Berlin publications lagged behind that of West-Berlin (Fig. 8; Table 1). The higher number of citations in 1960 and 1964 can be attributed to the high prevalence of cardiovascular disease in Eastern Europe (Heinemann et al. 1995; Ginter 1997; Sans et al. 1997; Dorr et al 2020). In contrast, cardiovascular morbidity was lower in Western Europe, and the GDR primarily focused on therapeutic options for cardiovascular diseases (Hampton 1994; Movsisyan et al. 2020). From 1965 to 1974, the HUB in East-Berlin received only four citations, indicating the diminished recognition of East-Berlin’s scientific contributions in both national and international spheres (Baker 1993; Gzoyan et al. 2023; Fig. 8).

Adopting English after 1969 significantly boosted citations and international recognition for West-Berlin publications (Gibbs 1995; Phillipson 2009; Bajerski 2011; Fig. 7). In 1972, citations in Berlin reached their peak (Fig. 7). Helmut Kewitz’s (1920–2009) research on choline concentration in the rat brain (rank 1) was the most frequently cited publication (original paper) from Berlin, with 168 citations (Table 1). Following closely were D. Neubert’s studies on the embryotoxic effects of 2,4,5-trichlorophenoxyacetic acid (rank 2) with 132 citations (Table 1). Both topics had relevance in the 1970s: Acetylcholine played a central role in Alzheimer’s research, and 2,4,5-trichlorophenoxyacetic acid garnered attention for its embryotoxic effects due to its use in the Vietnam War (Marchbanks 1980; Hess and Herring 1980; Dwernychuk et al. 2002).

In conclusion, the citation disparity between East-Berlin (3.3 citations per article) and West-Berlin (8.5 citations per article) underscores the profound impact of the political divide on national and international recognition in post-war Berlin (Narin et al. 1983; Fig. 9).

### Authors and affiliations

Examining post-war pharmacological research in Berlin unveils a narrative closely tied to political influences, as detailed in Tables 2 and 3. The Cold War era brought about a split in scientific activity and recognition between East- and West-Berlin. There were only very few scientific collaborations between East-Berlin and West-Berlin; all of them in the 1950s, before the erection of the Berlin wall (Table 2). Remarkably, one of these few papers (15 in Table 1) made it to the top-cited items of the Cold War, showing that excellent science is recognized across ideological borders. In contrast to East-Berlin, West-Berlin saw a marked expansion of the number of pharmacological research institutes (Table 3).

**Table 2:**
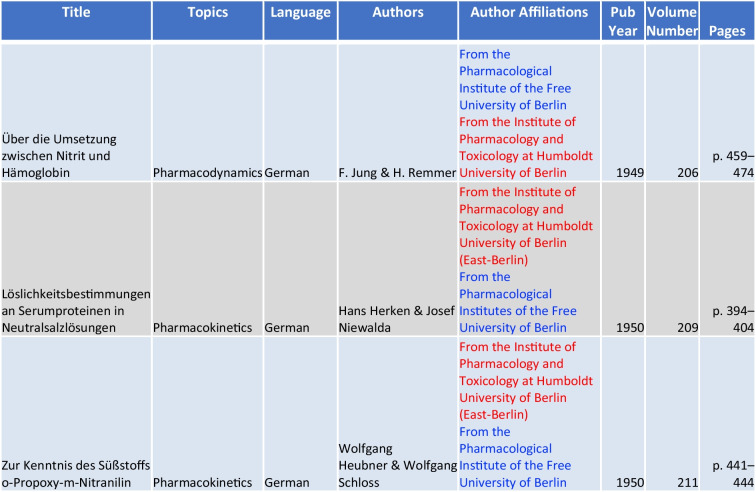
Collaborative publications between East- and West-Berlin Pharmacological Institutes—West-Berlin (blue) and East-Berlin (red)

**Table 3:** Founding Years of Pharmacological Institutes in West-Berlin (Philippu 2004)

Pharmacological Institutes of the Free University of Berlin	Year of foundation
Institute of Pharmacology, Free University of Berlin (FUB)	1948
Institute of Pharmacology and Toxicology, Veterinary Medicine, Free University of Berlin (FUB)	1951
Institute of Neuropsychopharmacology, Free University of Berlin (FUB)	1967
Institute of Clinical Pharmacology at the Steglitz Clinic (later Benjamin Franklin University Hospital) (FUB)	1969
Institute of Toxicology and Embryopharmacology, Free University of Berlin (FUB)	1972

**Fig. 10:**
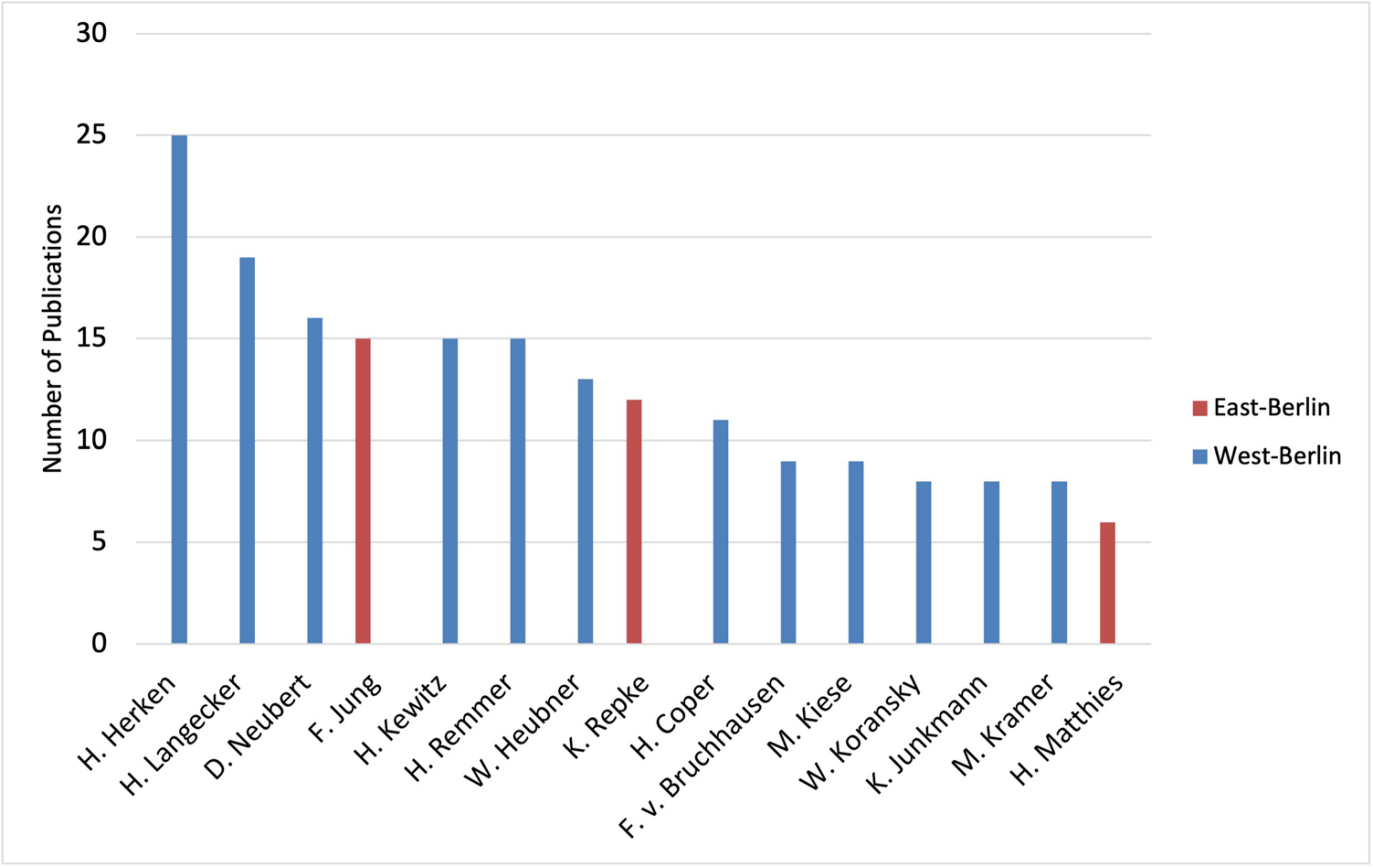
Publication output (original papers) of top 15 Berlin-based authors (1947–1974): West-Berlin (blue) and East-Berlin (red)

Central to Berlin’s pharmacological cold-war history is Hans Herken, whose academic contributions are evidenced by 25 original papers, as detailed in Table 4 and Fig. 10. Herken’s initial investigations into hunger edema evolved into studies on the pharmacodynamics and kinetics of diuretics, addressing prevalent health topics of the era (Herken 1999).

**Table 4:**
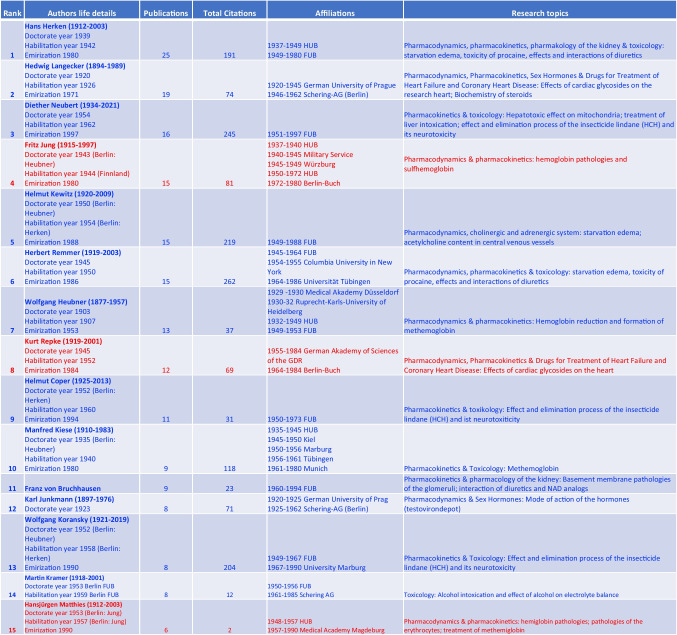
Top 15 authors’ scholarly impact (original papers) from West-Berlin (blue) and East-Berlin (red) (Bettendorf 1995, pp. 272; Philippu 2004, pp. 47-91, 99-112, 573, 574; Hartkopf 1992, p. 421). Authors are ranked accrding to their numbers of original publications

Following the destruction of the Friedrich-Wilhelms-Universität in 1949 (renamed as Humboldt University after World War II) and the relocation of its pharmacological institute to Dahlem, a resurgence in research was marked by Herken’s appointment as the head of the institute. This reconstruction era set the stage for the establishment of the FUB in December 1948, creating a stronghold of academic liberty in contrast to East-Berlin’s isolation under Soviet occupation (Candilis et al. 1999; Herken 1999; Philippu 2004).

Fritz Jung’s tenure at HUB from 1950 to 1972 was characterized by collaboration transcending ideological barriers, an extraordinary achievement during the Cold War era (Herken 1999; Philippu 2004). Despite significant political hurdles, Jung successfully nurtured cooperative relationships with colleagues at FUB, such as H. Remmer (Jung and Remmer 1949; Herken 1999; Table 2). The modest output of eight original papers from HUB between 1950 and 1962 (Fig. 1) mirrors the constrained research environment (Herken 1999; Vom Bruch et al. 2006).

Hansjürgen Matthies, a student of Jung, continued the scholarly lineage at HUB. His six publications, primarily dedicated to hemoglobin disorders, signify the academic perseverance within East-Berlin’s isolated confines (Hartkopf 1992; Philippu 2004).

The academic achievements are evident in the professional trajectory of Herbert Remmer, whose work at FUB is marked by 15 publications focusing on the pathophysiology of hemoglobin (Fig. 10). Remmer’s substantial research contributions at the FUB underscore the conducive research environment prevailing at the institute during that period (Jung and Remmer 1949; Herken 1999).

The collaboration within FUB is evident in the extensive body of work by Wolfgang Koransky and Helmut Coper. Their research on neurotoxicology and insecticide pharmacokinetics at the FUB highlights the institution’s pivotal role in advancing research areas that connect basic science with clinical applications (Philippu 2004; Oehme 2006). Hedwig Langecker from Schering AG is a prime example of a prolific industry pharmacologist from West-Berlin with a broad portfolio of research interests, ranging from pharmacodynamics to sex hormones. The latter field became a major business area of Schering AG. Langecker is the only prominent female pharmacologist in the Cold War era; otherwise dominated by men. 

**Fig. 11:**
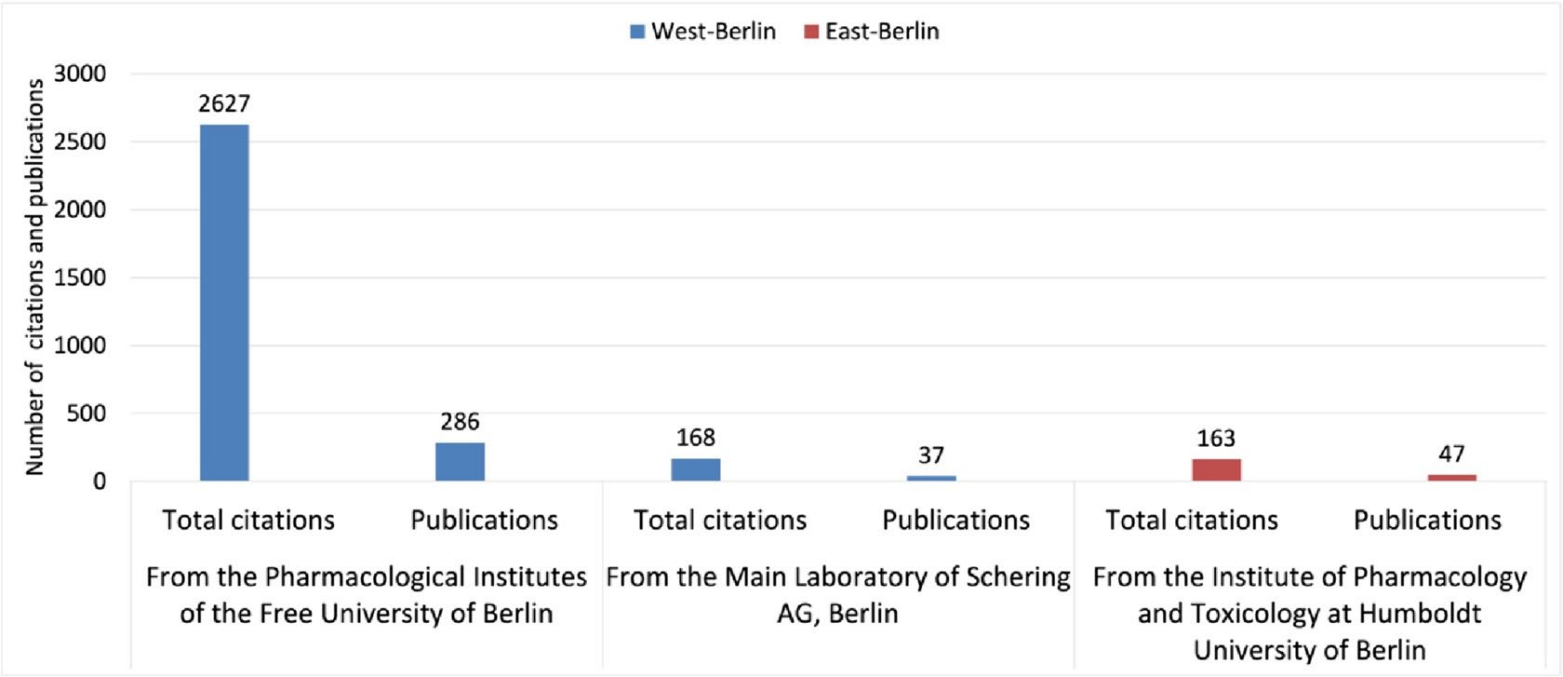
Institutional research impact: Pharmacological publications and their citations at Berlin’s Key Scientific Institutes (last accessed 10th March 2024)—West-Berlin (blue) and East-Berlin (red)

In contrast to West-Berlin, the situation of East-Berlin is best characterized by academic perseverance despite isolation (Fig. 11). Kurt Repke’s work at the Academy of Science of the GDR signifies a commitment to advancing cardiovascular pharmacology despite the obstacles posed by the Cold War (Bielka 2002; Philippu 2004).

The school of Berlin pharmacologists can be traced back to Wolfgang Heubner, under whose mentoring a cohort of influential scholars—Herken, Neubert, Jung, Kewitz, Remmer, Coper, Kiese, and Koransky-, emerged (Gerabek 2005; Vom Bruch et al. 2010). Their collective footprint in pharmacological research underscores not only Heubner’s pedagogical legacy but also his enduring impact on the scientific endeavors of his scholars (Starke 1998; Philippu 2004).

The role of Schering AG during post-war Berlin was pivotal, not merely surviving but adapting and thriving amid the ruins (Philippu 2004; Wlasich 2011). Their 37 publications in the post-war period attest to an industrial-academic dynamism that outpaced even the HUB institutions in citations (Fig. 11).

The pharmacological sector at the FUB significantly expanded with the incorporation of all pharmacological staff from HUB’s Veterinary Medicine to form the Institute of Pharmacology and Toxicology of Veterinary Medicine at the FUB in 1951 (Table 3). Helmut Kewitz, directing this institute from 1962 to 1969, contributed three original papers and later led the Institute of Clinical Pharmacology at the Steglitz Clinic from 1969. The Institute of Neuropsychopharmacology, directed by H. Coper until 1994, yielded two original papers, while D. Neubert's leadership of the Institute of Toxicology and Embryopharmacology from 1972 until 1997 also resulted in two papers by 1974 (Philippu 2004; Tables 3 and 4)

This analysis collectively refers to these entities as the pharmacological institute of the FUB to simplify, yet each played a pivotal role in West-Berlin’s pharmacological research during a transformative era. In summarizing the contributions and affiliations of the top 15 authors within Berlin’s pharmacological institutes, Table 4 offers a testament to the West’s dominance in scholarly output and citations.

### Topics

**Fig. 12:**
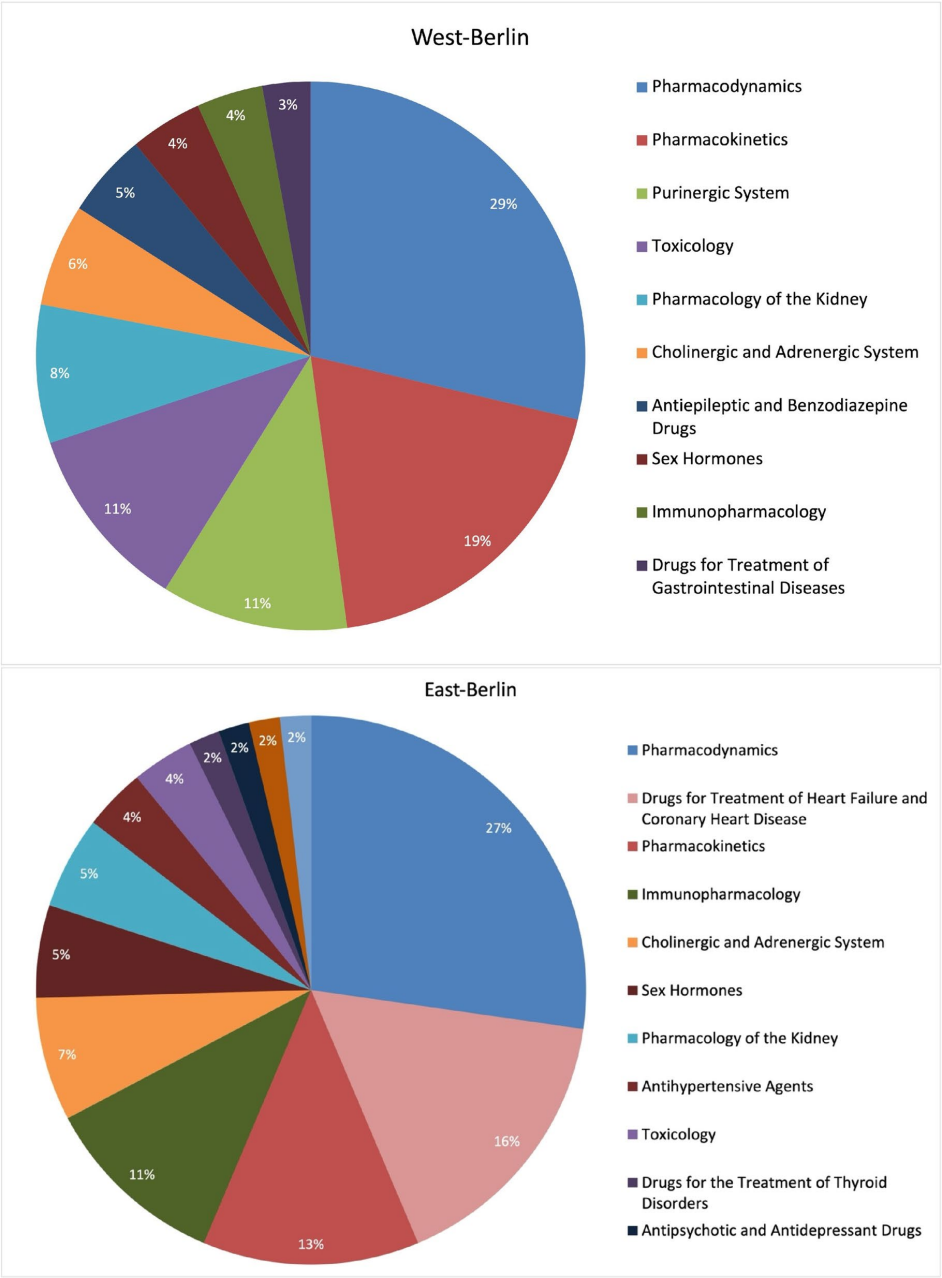
Comparison of the pharmacological research topics in East-Berlin and West-Berlin (1947–1974)

Pharmacological institutes of West-Berlin heavily worked on pharmacodynamics and pharmacokinetics (Fig. 12). These studies encompass various fields, for example, research on diuretics intersects both pharmacokinetics and renal pharmacology. The increase in pharmacodynamics and pharmacokinetics research during the early post-war years stemmed from the need for a more nuanced understanding of drug action and metabolism (Hochhaus et al. 2000; Takimoto 2001).

The prominence of pharmacodynamics and pharmacokinetics, can be attributed in part to the contributions of Heubner and Kewitz (Dross and Kewitz 1972; Philippu 2004; Fig. 12). Their research, spanning from methemoglobin to the effects of opiates on the gut and drug elimination, laid the groundwork for pharmacokinetics and served as a foundation for the development of new pharmacological treatments. This is exemplified in the case of starvation edema studied by Herken and Remmer (Herken et al. 1950; Herken 1999).

Toxicology was significantly influenced by Kramer’s work on the effects of alcohol and the research group led by Neubert, Coper, and Koransky on the neurotoxicology of insecticides (Matsumura 1985; Philippu 2004). Their contributions to the mechanisms of liver toxicity and specific substances such as HCH (γ-hexachlorocyclohexane) and procaine strengthened the understanding of toxicity at the cellular level and the importance of safety studies for pharmaceuticals (Philippu 2004; Fig. 13).

Publications on the purinergic system focused on NAD metabolism, antimetabolite effects, and the modulation of cyclic nucleotides (Schultz and Senft; Table 1 and Fig. 12). The research by Koransky, Neubert, Coper, and Herken on the involvement of ATP and ADP in the central venous vascular system, insulin interactions, and the impact of thiazide diuretics on cAMP degradation significantly contributed to our understanding of metabolic pathways and their pharmacological regulation (Bertrand et al. 1986; Philippu 2004).

Research in renal pharmacology focused on the metabolic effects of thiazides and the interaction of diuretics with insulin, deepening our understanding of renal physiology and pathophysiology (Herken et al. 1956; Philippu 2004).

The investigations on the cholinergic system by Kewitz and Neubert emphasize the importance of synaptic transmission and cholinesterase activities in understanding neuropharmacology (Dross and Kewitz 1972). Studies by Remmer on pharmacokinetic tolerance to phenobarbital and Neubert on the effects of hexobarbital highlight the significance of antiepileptic medications in modulating central nervous functions (Remmer et al. 1962).

In West-Berlin, research systematically addressed fundamental pharmacological principles and their clinical implications (Trendelenburg 1998; Herken 1999; Krige 2006).

In contrast, research in East-Berlin centered on pharmacodynamics and the treatment of heart failure and coronary heart disease (Fig. 12). Key advancements in pharmacodynamics were spearheaded by Jung and Matthies, who explored erythrocyte metabolism, methemoglobin reduction, as well as the effects of irritants and hemotoxins such as phenylhydrazine (Matthies et al. 1955; Matthies 1957). Treatment of heart failure and coronary heart disease, emerged as the second most prominent focus area (Fig. 12). The Cardiovascular Central Institute at the Academy of Science of the GDR, where researchers like Repke and Matthies worked, significantly contributed to the development of cardiovascular drugs (Philippu 2004; Timmermann et al. 2005).

The heightened prevalence of cardiovascular diseases in Eastern Europe and East-Berlin, acknowledged by health authorities under Soviet influence, prompted focused scientific inquiries into effective treatments, demonstrating a concerted endeavor to tackle these health issues (Heinemann et al. 1995; Ginter 1997; Dorr et al. 2020).

## Limitations and further studies

This analysis of Naunyn-Schmiedeberg’s Archives of Pharmacology, a journal with a distinctly Western orientation, offers only a partial view of the scientific endeavors in East-Berlin and East-Germany. It does not fully represent the scientific contributions and advancements of East-German pharmacologists and institutions, as it primarily highlights work recognized in the West. To gain a more comprehensive understanding of scientific productivity in East-Germany during the Cold War, future studies should encompass a broader range of journals, particularly those originating from the GDR and other socialist countries. An example of such a journal is “Die Pharmazie,” a significant pharmacological journal founded in the GDR in 1946 (Friedrich and Helmstädter 2020). A broader approach could unveil overlooked or politically marginalized scholarly work, thereby enhancing the recognition and appreciation of research achievements that have been underrecognized due to political divisions of the era.

## Conclusions

This bibliometric analysis of Naunyn-Schmiedeberg's Archives of Pharmacology (1947–1974) illuminates the division between East- and West-Berlin in the realm of pharmacological research during the Cold War. While West-Berlin and West-Germany, enjoying political freedom and international cooperation, dominated publication activities, institutes in East-Berlin faced political isolation and a constrained research environment (Herken 1999; Krige 2006; Tsvetkova 2008). This isolation was further compounded by the Western orientation of Springer-Verlag, limiting the visibility and integration of East-German pharmacologists into the international scientific community (Sarkowski and Götze 1992; Götze 1994; Starke 1998).

When constrained by ideologies, medical sciences, may reach their limits. This is here exemplified for pharmacology. This contrasts with areas like military and space science, which can sometimes flourish under these conditions. (Glanzel 2001; Bukkvoll 2015).

In summary, this case study reveals the stark impact of political context on Berlin’s pharmacological research during the Cold War. This paper demonstrates how political freedom, financial support, and internationalization boosted research productivity in West-Berlin. In contrast, political suppression, financial scarcity, and restricted international ties hindered scientific development in East-Berlin.

## Data Availability

All source data for this study are available upon reasonable request from the authors.

## Abbreviations

DGPT: Deutsche Gesellschaft für Experimentelle und Klinische Pharmakologie und Toxikologie (German Society for Experimental and Clinical Pharmacology and Toxicology)
SN: Serial number (in context of publication types)
DOI: Digital object identifier
CrossRefAn: Official DOI registration agency
Pandas: An open-source data analysis and manipulation tool in Python
LangdetectLanguage: Detection library for Python
BeautifulSoupA: Python library for pulling data out of HTML and XML-files
FUB: Free University of Berlin
HUB: Humboldt University of Berlin
FRG: Federal Republic of Germany
GDR: German Democratic Republic
USC: Unitarian Service Committee
